# Are ABO Blood Groups or Rh Antigen Perinatal Factors Affecting the Pass Rate of Transient Otoacoustic Emissions Screening Tests in Healthy Newborns during the First 48 h of Life?

**DOI:** 10.3390/ijns5010004

**Published:** 2019-01-04

**Authors:** Jose Miguel Sequi-Canet, Jose Miguel Sequi-Sabater, Jose Ignacio Collar-Castillo, Nelson Orta-Sibu

**Affiliations:** Department of Pediatrics, Hospital Francesc de Borja, 46701 Gandia, Spain

**Keywords:** ABO blood group, Rhesus antigens (Rh), otoacoustic emissions, hearing screening

## Abstract

Most hospitals recommend performing neonatal hearing screening. Transient evoked otoacoustic emission (TEOAE) tests represent an ideal technique for conducting this process. Previous studies have related the influence of ABO blood group and Rhesus antigens (Rh) on the susceptibility to various pathologies. However, available data about the potential relationship between ABO blood groups, Rh, and TEOAE pass rates are sparse. Recently, several authors concluded that O blood group and Rh+ are possible influential factors of TEOAE pass rates. Significantly different TEOAE amplitude response between the four main ABO blood groups were observed among normal-hearing individuals. Moreover, ABO blood groups were discussed as a possible influential factor for the development of noise-induced hearing loss later in life. The aim of this study was to investigate the relationship between ABO blood groups, Rh, and the first TEOAE pass rates in healthy newborns. Data were retrospectively collected from healthy newborns at the maternity ward of F. Borja hospital in Gandia (Spain). Rh and ABO were compared with the results of TEOAE performed within the first 48 h of life. Results: the study group included 2765 newborns. No significant correlation between ABO blood group or Rh and TEOAE pass rates was observed in comparative tables. Conclusion: ABO blood group and Rhesus antigens do not appear to have a significant impact on the pass rate of TEOAE screening in healthy newborns.

## 1. Introduction

ABO blood group system antigens are complex carbohydrate molecules located on the extracellular surface of red blood cell membranes. However, along with their expression on red blood cells, ABO antigens are also pervasively present on a variety of human cells and tissues, including the epithelium, sensory neurons, platelets, and the vascular endothelium surfaces. The clinical significance of the ABO blood group system extends beyond transfusion medicine; several reports suggest an important involvement in cardiovascular, oncological, and other diseases [1].

In 1985, Mollicone et al. [2] described the expression of B and H antigens on the primary sensory cells of the rat’s inner ear, highlighting a possible relation between blood group and auditory function. The appearance and progressive disappearance of B and H antigens on sensory and non-sensory cells can be correlated with significant events in the development of the cochlea. Previous observations suggest that human blood-group antigen expression on developing cochlear hair cells of rats may be related to afferent nerve fiber influence. Additionally, the temporal expression of H blood group antigen was discussed to be related to stereocilia differentiation in the development of cochlear hair cells. Moreover, thyroxine was observed as a possible modulator of blood-group antigen expression influencing cochlear hair cell development [3,4,5,6].

Otoacoustic emissions (OAEs) are low-level acoustic signals generated in the cochlea and passed through the middle ear into the external ear canal. OAEs are an objective indication of normal cochlear function. OAEs occur in nearly all ears with normal hearing and middle ear function, with larger amplitude found in females and in right ears. Additionally, OAEs can be classified into spontaneous OAE (SOAEs) and evoked OAEs. SOAEs arise in normal-hearing ears independently without external acoustic stimulation; SOAEs exist in 50–70% of ears with normal hearing. Detecting SOAEs alone is not clinically useful, but, on average, ears with the presence of SOAEs exhibit larger amplitude evoked OAEs than do ears without SOAEs. Evoked type OAEs include transient-evoked otoacoustic emissions (TEOAEs) and distortion-product otoacoustic emissions (DPOAEs). Both are clinically useful and often selected for monitoring cochlear function. TEOAEs and DPOAEs have been shown to have good sensitivity and good specificity for hearing screening. TEOAEs are measured after a broadband click is presented to the ear. TEOAEs are evoked across a broad frequency range when stimulated by temporally short clicks, which have broad spectral bandwidths, and multiple cochlear locations may contribute to TEOAEs measured at any specific frequency. DPOAEs are sounds generated in the cochlea in response to two primary tones simultaneously presented to the ear canal of the same ear. DPOAE testing can record evoked OAEs of higher frequencies than TEOAE testing. Like TEOAEs, DPOAEs are measurable in almost all normal-hearing ears without middle ear pathology, remaining relatively stable over time. Researchers have compared the sensitivity of evoked OAE testing with pure-tone audiometry and concluded that OAE testing is more sensitive in detecting the early onset of cochlear pathologies before a change in hearing thresholds [7].

Newborn hearing screening is both crucial and routinely performed. Only 50% of babies born with hearing loss have a hearing loss risk factor. Early detection leads to timely treatment of the affected neonates, resulting in a better final prognosis [8,9,10].

Several techniques are used in newborn hearing screening. Transient evoked otoacoustic emission (TEOAE) testing is one of the most frequently used techniques because of its accuracy, simplicity, speed, and low cost. Additionally, as previously stated, TEOAE testing offers high sensitivity and specificity, especially regarding cochlear status in hearing loss [11,12].

A major drawback of TEOAE testing as a screening technique for newborns relates to the middle ear status, which can severely affect its pass rate. Also, the presence of debris and vernix in the external meatus of the newborn can result in false positive screenings. Both factors lead to a failure rate greater than the actual hearing loss, thus a two-step newborn hearing screening protocol has been introduced to allow for clearance of the middle and external ear.

There are well-known hearing loss risk factors defined by the Joint Committee on Infant Hearing and The Commission for Early Detection of Hearing Loss (CODEPEH) based in Spain [10]. However, some studies have demonstrated that other epidemiological factors, such as gender or breastfeeding, are also important elements in the TEOAE pass rate, acting as minor risk factors. Research continues to determine related hormonal and/or neural effects in the case of gender, or for earlier opening of the Eustaquian tube and/or middle ear clearance in the case of breastfeeding. The real response and pass rate of the TEOAE screening test must be taken into account as more data on these phenomena are gathered [13,14,15].

Additionally, another crucial factor is the newborn’s age at the time of testing, which can be related to the mother’s length of hospital stay depending on the type of birth (cesarean section vs. vaginally) [16]. Data strongly suggest that the prime testing window is beyond 24–48 h of life, as fluid in the middle ear and in the external meatus is normally significantly reduced by the second day of life. The average hospital stay in Spain for mothers after a vaginal delivery is 48 h, thus allowing for the implementation of a successful hearing screening program.

Recent studies [7,17] have suggested that blood groups O and Rh+ play a role in cochlear status, which may result in a poorer response to the otoacoustic emissions test. This raises the question of whether these factors can influence the TEOAE screening results, like other previously described epidemiological factors, such as gender, have shown to incide in the TEOAE pass rate.

The findings contrast with other authors who concluded that blood group O (and possibly AB) plays a preventive role, while blood group A or B are burdensome for otitis media with effusion (OME) which can affect positively amplitude of TEOAE due to normal middle ear status allowing better OAE detection. No relationship was found when examining Rh factor as a predictor for OME [18].

Until now, these human studies related to risk of hearing loss and blood group type or Rh are mainly in adults, whilst no study provides an exploration of blood groups or Rh in relation to congenital hearing status. That is the reason why, in order to minimize the potential for data bias or unclear results, this research study focused on analyzing information exclusively on healthy newborns vaginally delivered without any hearing risk factors.

## 2. Materials and Methods

Significant differences in TEOAE amplitudes between groups can alter the pass rate of screening tests; therefore, the aim of this study was to analyze ABO blood group and Rh influences on the pass rate of the TEOAE test as a method for universal newborn hearing screening during the first 48 h of life.

Data were collected between 2016–2018 from all healthy newborns without known hearing loss risk factors in the maternity ward of the Francesc de Borja Hospital in Gandia (Spain). The study was approved by the ethical committee of this Hospital on 31 January 2018 with code e2/2018.

### 2.1. Exclusion Criteria

The focus of the study was limited to healthy newborns. Neonates who were admitted to the neonatal center exhibiting symptoms of any disease were not included in the research. Additionally, newborns with Apgar lower than 7 at 5 min were excluded, as were newborns delivered by caesarean section based on the practice of performing otoacoustic emissions after 72 h of birth. As previously stated, it is widely known that time has a positive influence on the initial test response.

### 2.2. Protocol

The bilateral TEOAE screening was performed as close to 48 h of life as possible. All nurses performed the screening, on every shift, every day of the week, depending on availability and work load. The screening was performed in a newborn room with as little background noise as possible after parental verbal consent was obtained. Testing usually took place after feeding time to ensure the newborn was calm. No sedation was administered.

### 2.3. Techniques

The TEOAEs were recorded with an ECHOCHECK OAE Screener^®^ based on the ILO88 (Otodynamics Ltd. Hatfield, U.K.) system and connected to the ILO ECP^®^ neonatal probe. The system utilizes a click-type non-linear stimulus of 1 ms duration, the intensity of which is 84 ± 3 dB sound pressure level (SPL) and 80 c/s frequency. A normal result (pass) requires a signal/noise level response above 6 dB with a frequency range from 0–6 kHz. A newborn with normal bilateral response was accepted as a pass; otherwise it was deemed a referral [19].

Determination of blood group and Rh was completed using neonatal cord blood with anticoagulant etilendiaminotetraacetic acid (EDTA) by means of DG Gel Newborn card (Grifols^®^, Barcelona, Spain).

The DG Gel Newborn card is a plastic support consisting of eight microtubes. Each microtube consists of a column and a dispensing/incubating chamber, while each column contains polymerized dextran microspheres in a buffered medium that acts as a filter. The dextrans are mixed with a reagent that contains specific antibodies, anti-human globulin, or a buffer.

The microtubes contain specific antibodies in the gel solution that act as a reaction medium and the red blood cells agglutinate in contact with the antibodies.

### 2.4. Statistical Analysis

The dependent variable is the first TEOAE result (pass/refer).

The independent variables are ABO blood group (group O/Other) and Rh (+/−) of newborn.

Following frequency analysis of the variables, a univariate analysis was completed between the TEOAE result and the study variables with the Chi-squared test and risk estimate with odds ratio.

Statistical analyses were only conducted for patients that had data available for either of the study variables (blood group O/other vs. TEOAE pass/refer or Rh +/− vs. TEOAE pass/refer). In order to minimize the risk of inaccurate data analysis, the relationships between gender and breastfeeding (well known factors influencing the TEOAE pass rate) versus blood groups and Rh groups were analyzed.

The significance level was established at *p* < 0.05. The data were analyzed using Excel^®^ 2016 and SPSS^®^ version 20.

## 3. Results

### 3.1. Blood Group Analysis

Data concerning ABO blood groups were available for 2765 newborns, of which the majority showed A and O blood group (Table 1). Based on previous observations suggesting that only the O blood group influences TEOAE pass rates, blood groups A, AB, and B were pooled to “others” for further exploration of the correlation between TEOAE and ABO blood groups (Table 2).

The relationship between blood group and TEOAE pass rate was not significant, as shown in Table 2.

There were no statistical differences with respect to gender or lactancy between blood groups (Table 3 and Table 4).

### 3.2. Rh Analysis

Rh was registered in 2765 newborns (386 Rh− vs. 2379 Rh+). Distribution is shown in Table 5.

Relationship between Rh and TEOAE pass rate was not significant (Table 6).

There were no Rh statistical differences between groups in gender or in lactancy (Table 7 and Table 8).

## 4. Discussion

Recent studies have concluded that there are some differences in TEOAE response in adults depending on blood groups or Rh; studies to determine if these differences are present in newborns that have not suffered ear injuries and are supposed to have intact hearing capacity (thus a normal TEOAE response) are absent. The results of the present study suggest there is not any influence between O blood group or Rh+ and results (pass rate) in the first TEOAE registered in healthy newborns vaginally delivered at 48 h of life; however there are some observations worth discussing.

### 4.1. ABO Blood Group

A recent study from Chow [17] stated that the amplitude response of otoacoustic emissions (OAEs) can be significantly different among normal-hearing individuals from the four main blood groups. Only 60 female participants were included in the study because of the consideration of the sex differences previously found in OAE measurements (better OAE in females) [14,15,16]; therefore, analyzing gender differences between blood groups in the present study as a variable was crucial. No differences were found between genders in blood groups (Table 3), thus results are reliable. In Chow’s study, nonlinear TEOAEs were recordable in the ears of participants with all of the blood groups—A, B, O, and AB. A one-way analysis of variance (ANOVA) was used to check for differences in TEOAE amplitudes among the four blood groups at frequencies 1.0, 1.4, 2.0, 2.8, and 4.0 kHz, in both right and left ears. No statistically significant differences were observed in these comparisons, but a trend of reduced amplitudes (although not statistically significant) for OAEs in blood group O compared with the three other blood groups was observed in TEOAE measurements at 1.0, 1.4, and 4.0 kHz in left ears and in TEOAE measurements at 1.4, 2.8, and 4.0 kHz in right ears. Participants with blood group B had a significantly higher prevalence of spontaneous OAEs (SOAEs) and larger distortion product OAE (DPOAE) response amplitude at certain frequencies compared with those with blood group O. The study showed that the participants with blood group O had statistically lower DPOAE amplitudes at 1001 Hz, 1257 Hz, and 1587 Hz, in left ears compared with those with other blood groups. Such findings suggested that there might be differences in nonlinear outer hair cell distortion mechanisms across blood groups. TEOAE are predominantly reflection-source emissions generated by acoustic scattering from intrinsic irregularities along the basilar membrane, while DPOAEs are generated mainly by distortion-source OAEs in addition to reflection-source OAEs. The differences in the generation mechanisms of TEOAEs and DPOAEs provide a possible explanation for the discrepancy in findings in amplitudes of TEOAEs and DPOAEs in the study. Chow suggested that individuals with blood group O might be genetically predisposed to features that affect OAE parameters. For instance, patients may have larger ear-canal volume and mass of the middle ear system.

In a similar study from Chen [7] with 60 male participants, nonlinear TEOAEs and linear TEOAEs were recorded in all four blood groups. Chen found that TEOAE as well as DPOAE amplitudes, at certain frequencies, varied significantly among the four blood groups, and blood group O individuals exhibited reduced OAE response amplitudes compared with non-O blood groups. Specifically, blood group O showed lower amplitudes of nonlinear TEOAEs in sum, on average, and at 2.0 kHz in the left ears, than participants with blood groups A and B; lower total signal to noise ratios (SNR) of nonlinear TEOAEs in the left ears than blood group B; and lower nonlinear TEOAE amplitude at 2.8 kHz in the right ears when compared with those with blood group AB. Linear TEOAEs generally allow higher SNR of TEOAEs and may thus increase observed OAE amplitude differences among the four blood groups. However, this was not noted in Chen’s study. While increasing the magnitude of TEOAE responses overall, statistically significant findings among the four blood groups were reduced. This is important because ECHOCHECK OAE Screener^®^ uses a nonlinear stimulus and thus increases the validity of our results, instead of being a limitation.

Similar findings were reported by Prabhu [20]. This study focused on determining if the blood group had any effect on high-frequency auditory sensitivity using ultrahigh-frequency audiometry and ultrahigh-frequency distortion product otoacoustic emissions (DPOAEs) in 60 females. The findings suggest that there was a significant reduction in DPOAE amplitude for individuals with blood group O versus individuals with other blood groups. However, there was no significant difference in ultrahigh-frequency thresholds across the blood groups. Prabhu [18] concluded that such phenomena may be attributed to a lower number of healthy outer hair cells in individuals with blood group O.

There may be a number of possible explanations for this observed occurrence. These studies were completed in adults; therefore, age or other factors may have influenced the hearing response. Additionally, perhaps individuals with blood group O might be genetically predisposed to factors that affect OAE parameters related to outer hair cell function.

Furthermore, noise-induced hearing loss was found to occur less in Turkish individuals with a non-O blood group (blood groups A, AB, and B) than those with blood group O [21], while a higher prevalence of noise-induced hearing loss (NIHL) was noted in Indian military personnel with blood group O [22]. These studies suggested that blood group may have a role in predicting individual risk of developing NIHL, but does not necessarily reflect congenital differences.

Susceptibility to noise-related hearing loss and the progression of damaging outcomes are highly variable among exposed individuals [23], and the development of NIHL can be influenced individually by, or by a combination of environmental, medical, and genetic risk factors. Blood group O individuals may have fewer outer hair cells (OHCs) or less active cochlear OHCs. It may be possible that these individuals are more prone to spontaneous hair cell loss at an earlier age. The biological differences among blood groups may provide evidence to help explain the apparent disadvantage of blood group O individuals in auditory status [24].

The low number of patients analyzed is a limitation of these studies, as is age (adult people with possible audiological injuries), thus opening the possibility of other epidemiological factors influencing the OAE response.

The data stemming from a robust number of newborns in this study strongly suggest that there are not any differences in the pass rate of TEOAE tests at birth (Table 3); it can be thus concluded that there are no significant differences in amplitude of response in the explored frequencies.

The findings also suggest that the blood group perhaps only expresses a risk factor for the development of hearing loss, increasing the susceptibility to common life injuries such as noise. It is also not a congenital hearing handicap in newborns and a less important factor to obtain a refer result in the TEOAE test.

Widely accepted data-altering factors, such as gender or breastfeeding, were not significantly different between groups (Table 3 and Table 4), thus helping to provide more reliable results.

### 4.2. Rh

The Rh system is one of the clinically important blood groups. Human blood group antigens are transiently expressed in developing cochlear hair cells. The temporal antigen expression seems to correspond to the main events of inner ear differentiation (e.g., hair cell development, synaptogenesis, ciliogenesis), relating inner ear characteristics to Rh.

Bener et al. [25] provided statistically significant results suggesting that more hearing loss is detected in Rhesus-positive babies compared with Rhesus-negative babies. Given the lack of historical research on this topic, the researchers concluded that this relationship required further detailed studies for verification purposes.

More recently, Aycicek [26] conducted a study in 438 male patients with a mean age of 43 years; the research demonstrated the existence of a significant relationship between Rh-positive groups and noise-induced hearing loss that may be associated with individual susceptibility, which could be dependent on the variety of Rh-associated glycoproteins. The researchers related this effect to clinical and experimental reports that have established a strong relationship between the kidney and the inner ear in a variety of modalities. Both organs contain similar anatomical and ultra-structural features related to their common physiological role in fluid and electrolyte balance. The two organs also share several common transporters/channels, and disturbances in the function of the inner ear and kidney based on either genetic predisposition or drug intervention (nephrotoxicity, ototoxicity), which may occur at the same time, affecting both organs.

As the Rh-associated glycoproteins play an important role in the ammonium transport in the kidneys, they might also have a similar role in the inner ear, which is anatomically and ultra-structurally similar.

This research study did not find any evidence on the difference in the pass rate of the TEOAE test related to Rh (Table 6). Perhaps other events can result in developing hearing loss, thus explaining the incidence of the phenomena in adults. Additional factors in newborns that alter TEOAE pass rate, such as breastfeeding or gender, were not significantly different between groups, thus providing reliable results (Table 7 and Table 8).

In summary, neither blood group nor Rh were important factors at birth to modify pass rate of TEOAE tests, an important question for newborn hearing screening programs. Differences stemming from adult studies must be further researched and confirmed given the lack of evidence to suggest congenital origin of the deficiency. It is thus necessary to study other factors that can influence the TEOAE response in adult life.

### 4.3. Limitations

The Echocheck Screener results do not provide actual TEOAE response amplitude values. As stated by other authors, it is possible that blood group O or Rh-positive newborns may have reduced amplitude OAE responses compared with the other blood groups. Although the responses may be significantly lower, those could still be adequate for newborns to pass the screening test. The TEOAE test without normal results indicates a hearing loss greater than 30 dB HL; therefore, given that there were no differences between groups, it can be stated that blood group does not make a significant difference in screening results. However, this study cannot conclude that there is not a difference in OAE response amplitudes across blood groups. Additional studies using actual response amplitude data are needed to consider the latter point.

The nonlinear protocol utilized in the current study is the most common method to record TEOAEs [19,27]. This method uses three clicks of one polarity with a subsequent single click with three times the amplitude and opposite polarity. The test can detect cochlear responses in the presence of linear artifacts related to the clicks. However, part of the actual OAE recording is eliminated as all linear components of the response are removed. Therefore, nonlinear measurement may not be able to detect the OAE response completely; this process results in a low signal-to-noise ratio of TEOAEs in general and may possibly contribute to the lack of amplitude difference among blood groups, thus not altering the pass rate. As discussed before, that issue was analyzed by Chen [7] and Chow [17], with controversial results regarding the relation between TEOAE amplitudes and blood groups. Perhaps it is necessary that linear measurement of TEOAEs should also be recorded in addition to using a nonlinear protocol in order to clarify this issue in future research.

The Echocheck Screener explores a frequency range from 0 to 6 kHz; previously cited studies showed some effect in 2 kHz in the range of Ecocheck. However, further studies are required to determine if there is any effect in some of the frequencies out of this range, such as differences in higher frequencies that cannot be detected with this device.

Given that healthy newborns were examined for this study, it remains unknown if blood group or Rh increases the susceptibility to other neonatal hearing loss factors such as hypoxia or ototoxic drugs adding risk and lowering TEOAE response in the newborns. The same limitation applies to newborns delivered by cesarean section that were excluded based on the TEOAE tests being registered after 48 h, when pass rate results are increasingly reliable.

Additionally, perhaps there are perinatal factors stronger than blood group or Rh that can mask their effect if the influence is mild in nature. This research study analyzed and accounted for well-known factors such as gender and breastfeeding, however, there may be other unknown elements that could influence results.

Failing the TEOAE test reflects hearing loss; however, there are other factors that can alter the results, including the high frequency of the etiology not being in the inner ear but in the middle ear or in the external meatus. The findings make it cumbersome to pair the test result to actual cochlear response, however, given the large sample size, and the fact that all the newborns in the study were healthy, it can be assumed that in order to pass the test, the influence of middle ear status was similar for all.

## 5. Conclusions

ABO blood group and Rhesus antigens (Rh) appear to not be significant factors in the pass rate of TEOAE screening in healthy newborns, however, additional studies are needed to determine if there is any mild effect in the response amplitude that is not significant enough to make a “test refer”, or if it affects frequencies not explored by the Echocheck screener.

## Figures and Tables

**Table 1:** The distribution of blood groups.

Blood Groups	Frequency	Valid Percent
A	1123	40.6
AB	153	5.5
B	356	12.9
O	1133	41.0
Total	2765	100.0

**Table 2:** Crosstab blood group O vs. other.

Blood Group	Frequency	Valid Percent		TEOAE		Total
				TEOAE REFER	TEOAE PASS	
O	1133	41	Count	118	1012	1130
			% within Group	10.4%	89.6%	100.0%
			% within TEOAE	38.4%	41.3%	41.0%
Other	1632	59	Count	189	1440	1629
			% within Group	11.6%	88.4%	100.0%
			% within TEOAE	61.6%	58.7%	59.0%
Total	2765	100	Count	307	2452	2759
			% within Group	11.1%	88.9%	100.0%
			% within TEOAE	100.0%	100.0%	100.0%
**Chi-Square Tests**	**Value**			**df**	**Asymp. Sig. (2-Sided)**	
Pearson Chi-Square	0.907			1	0.341	
**Risk Estimate**				**Value**	**95% Confidence Interval**	
					**Lower**	**Upper**
Odds Ratio for Group (O/Other)				0.888	0.696	1.133
For cohort TEOAE REFER				0.900	0.724	1,118
For cohort TEOAE PASS				1.013	0.987	1.040
*N* of Valid Cases				2759		

TEOAE—transient evoked otoacoustic emission; df—degrees of freedom.

**Table 3:** Crosstab gender vs. blood group.

Gender		Group		Total
		O	Other	
Female	Count	567	795	1362
	% within Gender	41.6%	58.4%	100.0%
	% within Group	50.0%	48.7%	49.3%
Male	Count	566	837	1403
	% within Gender	40.3%	59.7%	100.0%
	% within Group	50.0%	51.3%	50.7%
Total	Count	1133	1632	2765
	% within Gender	41.0%	59.0%	100.0%
	% within Group	100.0%	100.0%	100.0%
**Chi-Square Tests**	**Value**	**df**	**Asymp. Sig. (2-Sided)**	
Pearson Chi-Square	0.474	1	0.491	
**Risk Estimate**		**Value**	**95% Confidence Interval**	
			**Lower**	**Upper**
Odds Ratio for Gender (Female/Male)		1.055	0.906	1.227
For cohort Group = O		1.032	0.944	1.128
For cohort Group = Other		0.978	0.919	1.041
*N* of Valid Cases		2765		

**Table 4:** Crosstab lactancy vs. blood group.

Lactancy		Group	Total	
		O	Other	
Formula	Count	244	361	605
	% within Lactancy	40.3%	59.7%	100.0%
	% within Group	27.4%	28.9%	28.3%
Breast	Count	647	886	1533
	% within Lactancy	42.2%	57.8%	100.0%
	% within Group	72.6%	71.1%	71.7%
Total	Count	891	1247	2138
	% within Lactancy	41.7%	58.3%	100.0%
	% within Group	100.0%	100.0%	100.0%
**Chi-Square Tests**	**Value**	**df**	**Asymp. Sig. (2-Sided)**	
Pearson Chi-Square	0.627	1	0.428	
**Risk Estimate**		**Value**	**95% Confidence Interval**	
			**Lower**	**Upper**
Odds Ratio for Lactancy (Formula/Breast)		0.926	0.764	1.121
For cohort Group = O		0.956	0.853	1.070
For cohort Group = Other		1.032	0.955	1.116
*N* of Valid Cases		2138		

**Table 5:** Distribution of Rhesus (Rh) groups.

Valid	Frequency	Valid Percent
Negative	386	14.0
Positive	2379	86.0
Total	2765	100.0

**Table 6:** Crosstab TEOAE vs. Rh.

Rh		OEA		Total
		TEOAE REFER	TEOAE PASS	
Negative	Count	44	341	385
	% within Rh	11.4%	88.6%	100.0%
	% within TEOAE	14.3%	13.9%	14.0%
Positive	Count	263	2111	2374
	% within Rh	11.1%	88.9%	100.0%
	% within TEOAE	85.7%	86.1%	86.0%
Total	Count	307	2452	2759
	% within Rh	11.1%	88.9%	100.0%
	% within TEOAE	100.0%	100.0%	100.0%
**Chi-Square Tests**	**Value**	**df**	**Asymp. Sig. (2-Sided)**	
Pearson Chi-Square	0.041	1	0.839	
**Risk Estimate**		**Value**	**95% Confidence Interval**	
			**Lower**	**Upper**
Odds Ratio for Rh (Negative/Positive)		1.036	0.738	1.454
For cohort TEOAE = REFER		1.032	0.764	1.393
For cohort TEOAE = PASS		0.996	0.958	1.035
*N* of Valid Cases		2759		

**Table 7:** Crosstab Gender vs. Rh.

Gender		Rh		Total
		Negative	Positive	
Female	Count	174	1188	1362
	% within Gender	12.8%	87.2%	100.0%
	% within Rh	45.1%	49.9%	49.3%
Male	Count	212	1191	1403
	% within Gender	15.1%	84.9%	100.0%
	% within Rh	54.9%	50.1%	50.7%
Total	Count	386	2379	2765
	% within Gender	14.0%	86.0%	100.0%
	% within Rh	100.0%	100.0%	100.0%
**Chi-Square Tests**	**Value**	**df**	**Asymp. Sig. (2-Sided)**	
Pearson Chi-Square	3.137	1	0.077	
**Risk Estimate**		**Value**	**95% Confidence Interval**	
			**Lower**	**Upper**
Odds Ratio for Gender (Female/Male)		0.823	0.663	1.021
For cohort Rh = Negative		0.845	0.702	1.018
For cohort Rh = Positive		1.028	0.997	1.059
*N* of Valid Cases		2765		

**Table 8:** Crosstab Lactancy vs. Rh.

Lactancy		Rh		Total
		Negative	Positive	
Formula	Count	84	521	605
	% within Lactancy	13.9%	86.1%	100.0%
	% within Rh	28.1%	28.3%	28.3%
Breast	Count	215	1318	1533
	% within Lactancy	14.0%	86.0%	100.0%
	% within Rh	71.9%	71.7%	71.7%
Total	Count	299	1839	2138
	% within Lactancy	14.0%	86.0%	100.0%
	% within Rh	100.0%	100.0%	100.0%
**Chi-Square Tests**	**Value**	**df**	**Asymp. Sig. (2-Sided)**	
Pearson Chi-Square	0.007	1	0.933	
**Risk Estimate**		**Value**	**95% Confidence Interval**	
			**Lower**	**Upper**
Odds Ratio for Lactancy (Formula/Breast)		0.988	0.753	1.297
For cohort Rh = Negative		0.990	0.783	1.251
For cohort Rh = Positive		1.002	0.964	1.040
*N* of Valid Cases		2138		
